# Insufficient radiofrequency ablation promotes the metastasis of residual hepatocellular carcinoma cells via upregulating flotillin proteins

**DOI:** 10.1007/s00432-019-02852-z

**Published:** 2019-02-28

**Authors:** Ning Zhang, Hui Li, Chengdong Qin, Dening Ma, Yiming Zhao, Weiping Zhu, Lu Wang

**Affiliations:** 1 Department of Hepatic Surgery, Fudan University Shanghai Cancer Center, 270 Dong-An Road, Shanghai, 200032 China; 2 Department of Abdominal Surgery, Linyi Tumor Hospital, 60 Ling-Yuan East Road, Linyi, 276000 China; 3 Department of Breast Surgery, Zhejiang Cancer Hospital, 1 Ban-Shan East Road, Hangzhou, 310022 China; 4 Department of Colorectal Surgery, Zhejiang Cancer Hospital, 1 Ban-Shan East Road, Hangzhou, 310022 China

**Keywords:** Hepatocellular cancer, Radiofrequency ablation, Metastasis, Flotillin, β-Catenin

## Abstract

**Purpose:**

Radiofrequency ablation (RFA) therapy has proven to be effective and feasible for early-stage hepatocellular carcinoma (HCC); however, rapid progression of residual tumor cells after RFA has been confirmed, but the molecular mechanisms of this phenomenon are poorly understood. This study evaluated the effect of the lipid raft proteins known as flotillins on the invasive and metastatic potential of residual HCC.

**Methods:**

The human HCC cell line HCCLM3 was used to establish insufficient RFA models in vivo and in vitro. Changes in cellular morphology, soft agar colony formation, motility, metastasis, and epithelial–mesenchymal transition (EMT) markers after insufficient RFA intervention in vitro and in vivo were detected by real-time PCR, western blotting, immunohistochemistry and transwell assays.

**Results:**

The results showed that flotillin-1 and flotillin-2 expression were upregulated in HCCLM3 cells following 45 °C heat treatment and in residual HCCLM3 xenografts cells after insufficient RFA. Knocking down flotillin-1 or flotillin-2 in HCCLM3 cells by shRNA significantly lowered insufficient RFA-induced tumor growth, EMT changes, and metastasis in vitro and in vivo. Furthermore, mechanism studies indicated that flotillins altered the EMT status and metastatic potential of heat-treated HCCLM3 cells by activating the Akt/Wnt/β-catenin signaling pathway.

**Conclusions:**

Our findings present new evidence that flotillins play a key role in the aggressive behaviors of residual cancer cells after insufficient RFA and provide new insights into the regulatory mechanism of Wnt/β-catenin signaling.

**Electronic supplementary material:**

The online version of this article (10.1007/s00432-019-02852-z) contains supplementary material, which is available to authorized users.

## Introduction

Hepatocellular carcinoma (HCC) is one of the most common malignancies and the third leading cause of cancer-related deaths worldwide (Siegel et al. 2015). Although a variety of therapies such as surgical resection, liver transplantation, interventional treatment, and systemic therapies are employed, HCC patients have low 5-year survival rates and high recurrence rates due to invasion and metastasis (Au and Frenette 2015; Forner et al. 2012; Schlachterman et al. 2015). A better understanding of the molecular mechanisms that regulate HCC invasion and metastasis is essential for developing novel prognostic and of therapeutic strategies for HCC.

Radiofrequency ablation (RFA) is a local therapy that is an alternative to surgery for HCC < 3 cm and is widely used due to its simplicity, safety, minimal invasiveness, repeatability, and short hospitalization (Lee et al. 2017; Tsukamoto et al. 2018). However, cases of rapidly progressing residual HCC after insufficient RFA have been reported to be on the rise, with post-RFA recurrent rates ranging from 3.2 to 26% (Lam et al. 2008; Shiina et al. 2012). Moreover, local recurrences after RFA show more invasive growth and vascular invasion, and less differentiation compared with tumors from patients without RFA. Until now, the underlying mechanisms behind this phenomenon were still less unknown.

Lipid rafts function as physical platforms for various molecules that are involved in a variety of biological processes by serving as organizing centers for assembling signaling molecules into functional complexes (Babuke and Tikkanen 2007). Flotillins are key components of lipid rafts and include two members: flotillin-1 (FLOT1) and flotillin-2 (FLOT2). Previous studies have shown that flotillins are closely associated with tumor development, invasion, and metastasis (Bodin et al. 2014; Deng et al. 2018; Liu et al. 2018). Moreover, FLOT1 and FLOT2 were recently reported to be overexpressed and associated with progression and poor survival in HCC, suggesting the possibility of their use as prognostic markers or therapeutic targets in HCC (Wang et al. 2017; Zhang et al. 2013). Nevertheless, the biological role and molecular mechanism through which flotillins alter the biology of residual HCC after insufficient RFA remains unclear.

In this study, we investigated FLOT1 and FLOT2 expression in insufficient RFA tumor tissues in vivo and in heat-treated HCC cells in vitro, and further explored the roles of flotillins in altering the metastatic potential of residual HCC cells.

## Materials and methods

### Cell culture

The human HCC cell lines HCCLM3 (a highly metastatic HCC line established at the Liver Cancer Institute, Zhongshan Hospital, Fudan University, Shanghai, China) and HepG2 cells (a modestly metastatic HCC line obtained from the Cell Bank of the Chinese Academy of Sciences, Shanghai, China) were maintained in Dulbecco’s modified Eagle’s medium (DMEM; Gibco BRL, Rockville, MD, USA) with 10% fetal bovine serum (FBS; Life Technologies, Carlsbad, CA, USA) and 100 U/mL penicillin at 37 °C in a humidified atmosphere containing 5% CO_2_ (Li et al. 2004).

### Vectors, retroviral infections, and transfection

The lentiviral vectors pGCSIL-GFP-shRNA-FLOT1, pGCSIL-GFP-shRNA-FLOT2 and pGC-FU-GFP-CTNNB1 were purchased from GeneChem Co., Ltd. (Shanghai, China). pGC-FU-GFP-CTNNB1 was transfected into HCCLM3 cells to overexpress β-catenin, and pGCSIL-GFP-shRNA-FLOT1 and pGCSIL-GFP-shRNA-FLOT2 were transfected into HCCLM3 cells to silence FLOT1 and FLOT2, respectively. pGCSIL-GFP and pGC-FU-GFP were used as controls.

### Soft agar colony formation assay

Soft agar colony formation was performed using the CytoSelect 96-Well Cell Transformation Assay (Cell Biolabs Inc., San Diego, CA, USA) according the manufacturer’s protocol. Briefly, a bottom layer of 1.2% agar solution was plated and solidified, and then a top layer of equal volumes 1.2% agar solution, culture medium, and cell suspension (1 × 10^3^ cells/well) was added. Culture medium (100 µL) was added to the top layer of the soft agar and replaced with fresh medium every 3 d. The cells were incubated for 6–8 days at 37 °C. Numbers of microscopically visible colonies (> 0.1 mm) were counted.

### In vitro heat treatment

HCCLM3 and HepG2 cells were seeded into 6-well plates at 5 × 10^4^ cells/well. After 24 h, the plates were sealed with Parafilm and submerged in a water bath set to the target temperature for 10 min. The target temperatures for HCCLM3 and HepG2 cells were 39 °C, 42 °C, 45 °C and 41 °C, 44 °C, 47 °C, respectively; the control temperature was 37 °C. The soft agar colony formation, and cell migration and invasion assays were performed 48 h after heat treatments.

### Cell migration and invasion assays

Cell migration and invasion were assessed by transwell assays (Corning Inc., Corning, NY, USA). Briefly, 8 × 10^4^ cells in serum-free DMEM were seeded into the upper chamber of each well of 24-well plates containing 8.0-µm pore size membranes. DMEM containing 10% FBS was added to the lower chamber of each well. After 48 h, cells that had reached the underside of the membrane were stained with Giemsa (Sigma-Aldrich, St. Louis, MO, USA), and then five randomly selected areas (200× magnification) per well were counted. Cell invasion assays were performed similarly, except 80 µL of Matrigel (0.8 mg/mL, BD Biosciences, Franklin Lakes, NJ, USA) was added to each well 6 h before cells were seeded onto the membranes.

### RNA extraction and real-time quantitative (RT-q)PCR

Total RNA was isolated using TRIzol reagent (Invitrogen, Carlsbad, CA, USA) and reverse transcribed to cDNA using the PrimeScript RT reagent kit (Takara, Shiga, Japan). SYBR Premix Ex Taq (Takara) was used for RT-qPCR according to the manufacturer’s instructions (Livak and Schmittgen 2001). Relative mRNA expression levels were calculated by the 2^− ΔΔCt^ method after normalizing to β-actin as an internal control. (16) Sequences of the RT-qPCR primers were as follows: FLOT1, forward: 5′-CCCATCTCAGTCACTGGCATT-3′ and reverse: 5′-CCGCCAACATCTCCTTGTTC-3′; FLOT2, forward: 5′-CCCCAGATTGCTGCCAAA-3′ and reverse: 5′-TCCACTGAGGACCACAATCTCA-3′; E-cadherin, forward: 5′-CCCGGGACAACGTTTATTACTATG-3′ and reverse: 5′-TCAGCCGCTTTCAGATTTTCA-3′; *N*-cadherin, forward: 5′-CACGCCGAGCCCCAGTAT-3′ and reverse: 5′-GCCCCCAGTCGTTCAGGTAA-3′; Vimentin, forward: 5′-CTCTCAAAGATGCCCAGGAG-3′ and reverse: 5′-GCACGATCCAACTCTTCCTC-3′; Snail, forward: 5′-TGCAGGACTCTAATCCAAGTTTACC-3′ and reverse: 5′-GTGGGATGGCTGCCAGC-3′; β-actin, forward: 5′-GCTCCTCCTGAGCGCAAG-3′ and reverse: 5′-CATCTGCTGGAAGGTGGACA-3′.

### Western blotting

Western blotting was performed as in previous studies using anti-FLOT1, anti-FLOT2 (Sigma-Aldrich), anti-E-cadherin, anti-*N*-cadherin, anti-Vimentin, anti-Snail, anti-β-catenin and anti-Cyclin-D1 antibodies (Abcam, Cambridge, UK) (Zhang et al. 2014a). Membranes were stripped and re-probed with anti-β-actin (Sigma-Aldrich) as a loading control.

### Xenograft mouse model

Male BALB/c nude mice (5-week-old, 18–20 g) were purchased from SLAC Laboratory Animal Co., Ltd. (Shanghai, China) and housed under specific pathogen-free conditions. The experimental protocol was approved by the Ethical Committee on Animal Experiments of the Animal Care Committee of Fudan University. Xenograft HCC models were established by orthotopic implantation of histologically intact tumor tissue into the nude mouse liver according to previous protocols (Sun et al. 1996). Two weeks after orthotopic implantation, the insufficient RFA operation was performed as follows: insufficient RFA mice underwent laparotomy to expose the tumor, then, considering the weight and volume of nude mice, RFA was performed at a lower energy protocol, in which the out power was 5 W and the duration was 30 s. This ensured the presence of residual cancer. Mice in the control group were sham-operated by inserting a needle electrode into the tumor without performing ablation. 5 weeks later, all mice were euthanized and tumor volumes were measured. Tumor volume was calculated according to the formula: volume (mm^3^) = (largest diameter × shortest diameter^2^)/2 (Zhang et al. 2014a).Then, tumors and lungs were placed in a 4% paraformaldehyde solution. Lung tissues were serially sectioned and stained with hematoxylin and eosin.

### Immunohistochemistry

Tumor tissues were fixed, embedded, and sliced into 5-µm thick sections. FLOT1, FLOT2, E-cadherin, *N*-cadherin, Vimentin and β-catenin immunohistochemical staining were performed as previously described. Staining results were viewed under a light microscope (Olympus, Tokyo, Japan) (Zhang et al. 2014a). Stained slides were scored by two investigators according to values obtained from IRS systems.

### Statistical analysis

Experimental data are presented as mean ± SD. First, the Kolmogorov–Smirnov test was used to determine the normality of the data in each group. Differences between two normal distribution groups were estimated using Student’s *t* test. Differences among three or more normal distribution groups were analyzed using ANOVA. Differences between non-normal distribution groups were analyzed using nonparametric analyses of Chi-square tests. All analyses were performed using SPSS v20.0 software (IBM. Armonk, NY, USA); a two-tailed *P* value < 0.05 was considered statistically significant.

## Results

### Insufficient RFA increased FLOT1 and FLOT2 expression in HCCLM3 cells in vivo and in vitro

**Fig. 1:**
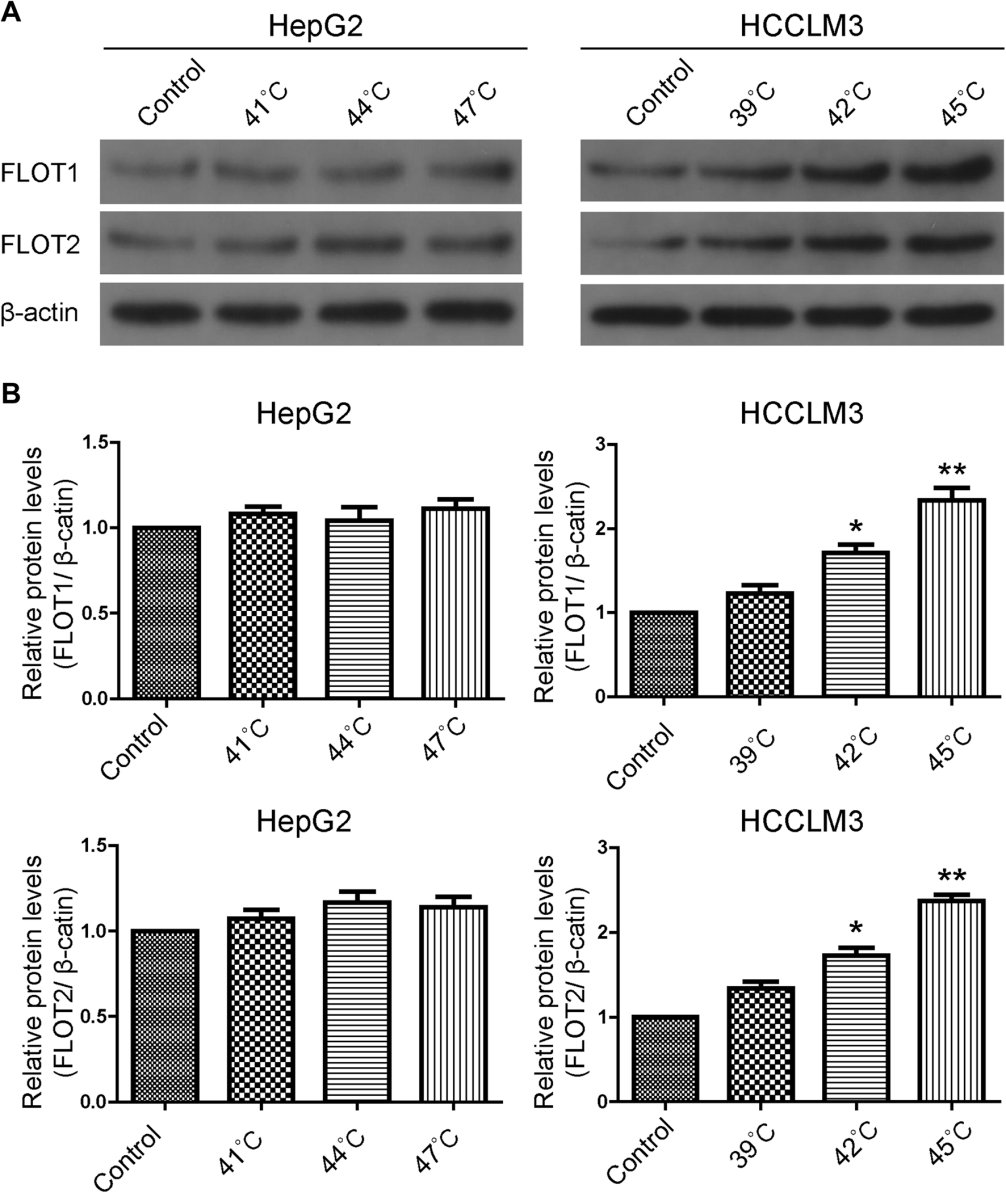
Insufficient RFA increased FLOT1 and FLOT2 levels in HCC cells in vitro. **a** Western blot analysis of FLOT1 and FLOT2 levels in HCC cells. HepG2 cells were lysed 24 h after 10-min heat treatments at 41 °C, 44 °C, and 47 °C, HCCLM3 cells were lysed 24 h after 10-min heat treatments at 39 °C, 42 °C, and 45 °C. **b** Densitometry analyses depict relative changes in FLOT1 and FLOT2 expression. Data are presented as mean ± SD. Experiments were independently conducted three times; *P < 0.05 and **P < 0.01

**Fig. 2:**
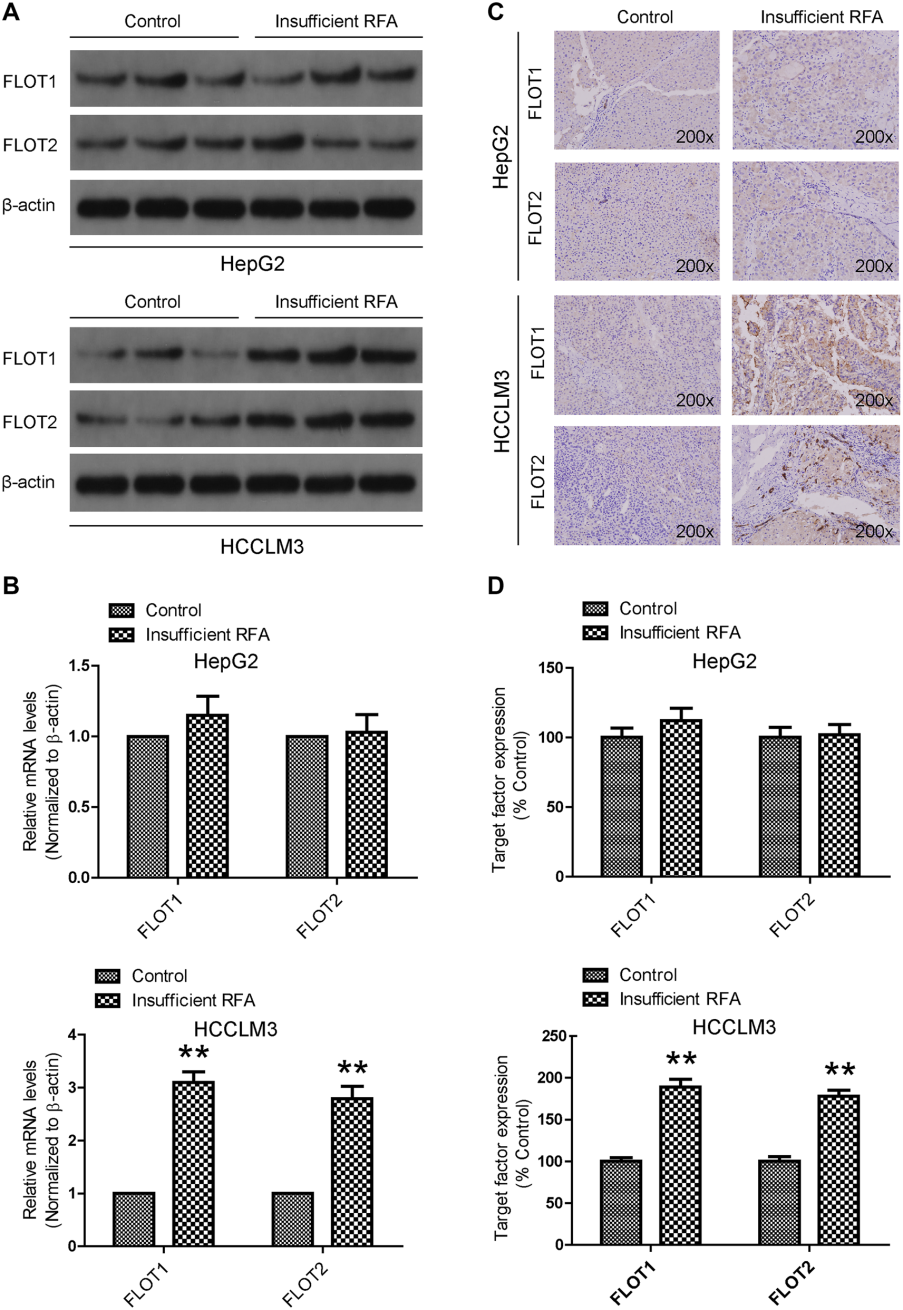
Insufficient RFA increased FLOT1 and FLOT2 levels in residual cancer in vivo. **a, b** FLOT1 and FLOT2 mRNA and protein levels in heat-treated HCCLM3 and HepG2 residual cancer and sham-operated tissue were detected by western blot and RT-qPCR. **c, d** Immunohistochemical staining confirmed increased FLOT1 and FLOT2 protein levels in heat-treated HCCLM3 residual cancer, but not in heat-treated HepG2 residual cancer. Representative images are shown at 200× magnification. Data are presented as mean ± SD. Experiments were independently conducted three times; *P < 0.05 and **P < 0.01

To examine the effects of insufficient RFA on FLOT1 and FLOT2 expression in vitro, expression was determined in HCC cells 24 h after 10 min heat intervention. Western blot results showed that FLOT1 and FLOT2 were significantly upregulated in heat-treated cells compared with the control group, especially at 45 °C for HCCLM3 cells. However, FLOT1 and FLOT2 expression were similar in heat-treated and control HepG2 cells (Fig. 1). Consistent with these in vitro results, western blot (Fig. 2a), RT-qPCR (Fig. 2b) and immunohistochemistry (Fig. 2c, d) results demonstrated that both FLOT1 and FLOT2 were significantly upregulated in insufficient RFA-treated HCCLM3 tumors compared with controls.

### Silencing FLOT1 and FLOT2 decreased the aggressiveness of heat-treated HCCLM3 cells in vitro

**Fig. 3:**
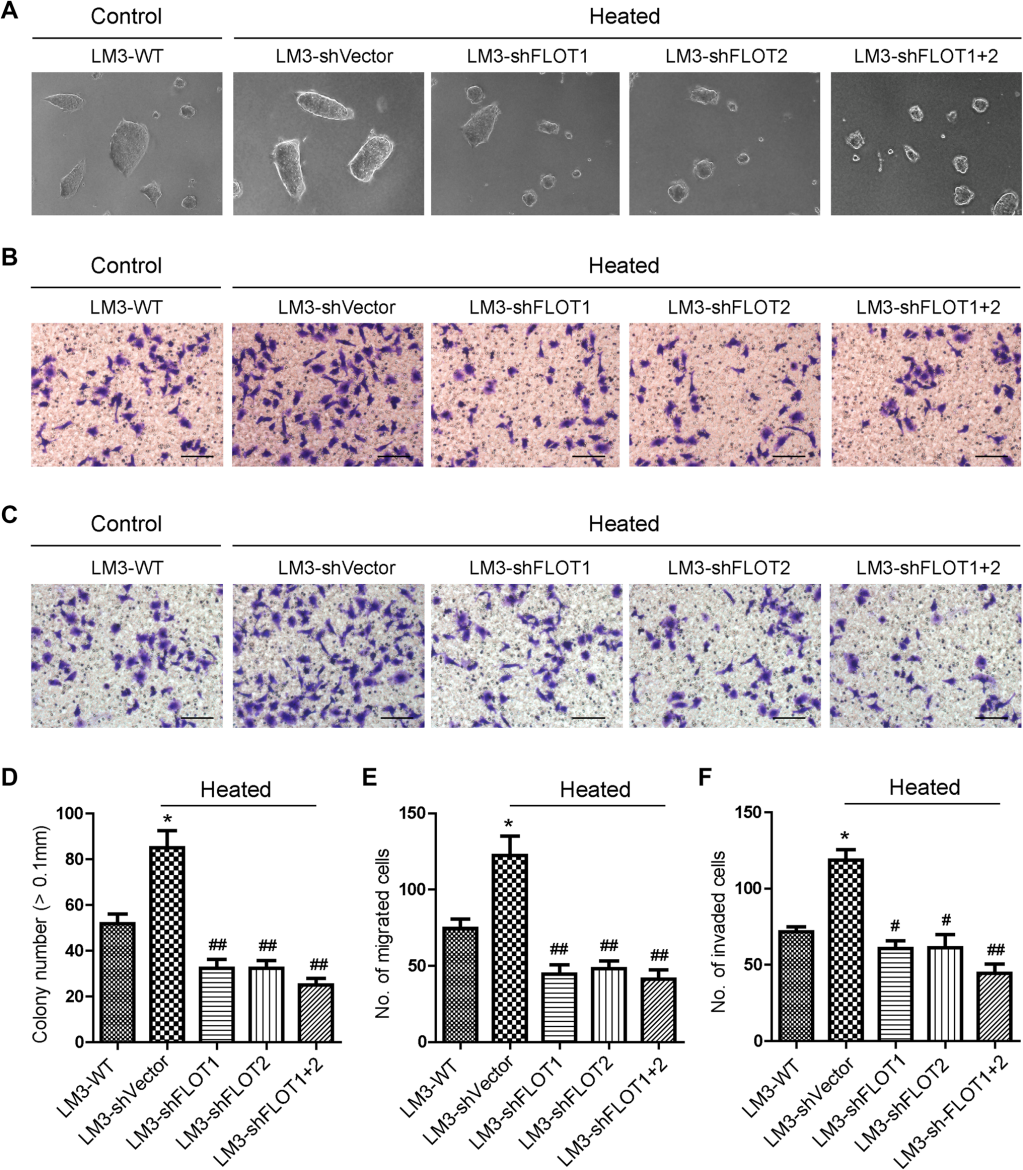
Silencing FLOT1 and FLOT2 decreased the in vitro aggressiveness of heat-treated HCCLM3 cells. **a, d** Representative images (**a**) and quantification (**d**) of HCCLM3 colony numbers from anchorage-independent growth assays. Colonies > 0.1 mm in diameter were scored. **b, e** Representative images (**b**) and quantification (**e**) of cell migration results of transwell assays; scale bar = 50 µm. **c, f** Representative images (**c**) and quantification (**f**) cell invasion results of transwell matrix penetration assays; scale bar = 50 µm. Data are presented as mean ± SD. Experiments were independently conducted three times; *P < 0.05, **P < 0.01 vs. the HCCLM3-WT group; ^#^*P* < 0.05, ^##^*P* < 0.01 vs. the heat-treated HCCLM3-shVector group

We further investigated whether FLOT1 and FLOT2 played a role in the increased metastatic capacity of HCCLM3 cells after insufficient RFA. When HCCLM3 cells were examined 24 h after 45 °C heat treatment for 10 min, soft agar colony formation assays showed that heat-treated HCCLM3 cells displayed higher anchorage-independent growth compared with control cells (Fig. 3a, d). Moreover, transwell migration (Fig. 3b, e) and matrigel invasion (Fig. 3c, f) assays revealed that heat treatment significantly increased the mobility and invasive capacity of HCCLM3 cells. Strikingly, silencing FLOT1, FLOT2, or with shRNA significantly reduced anchorage-independent growth, mobility, and invasion (Fig. 3a–f). Furthermore, the silencing FLOT1 or FLOT2 or both also reduced the anchorage-independent growth, mobility and invasive capacity of HCCLM3 cultured in 37 °C (Supplemental Fig. 1). Collectively, the above findings demonstrated that up-regulated expression of FLOT1 and FLOT2 was functionally relevant to invasion and metastasis of HCCLM3 cells mediated by heat intervention. Interestingly, the expression of FLOT1 and FLOT2 also differs in clinical specimens with different metastatic potential (Supplemental Fig. 2).

### Silencing FLOT1 and FLOT2 attenuated epithelial–mesenchymal transition (EMT)-like changes in heat-treated HCCLM3 cells in vitro

**Fig. 4:**
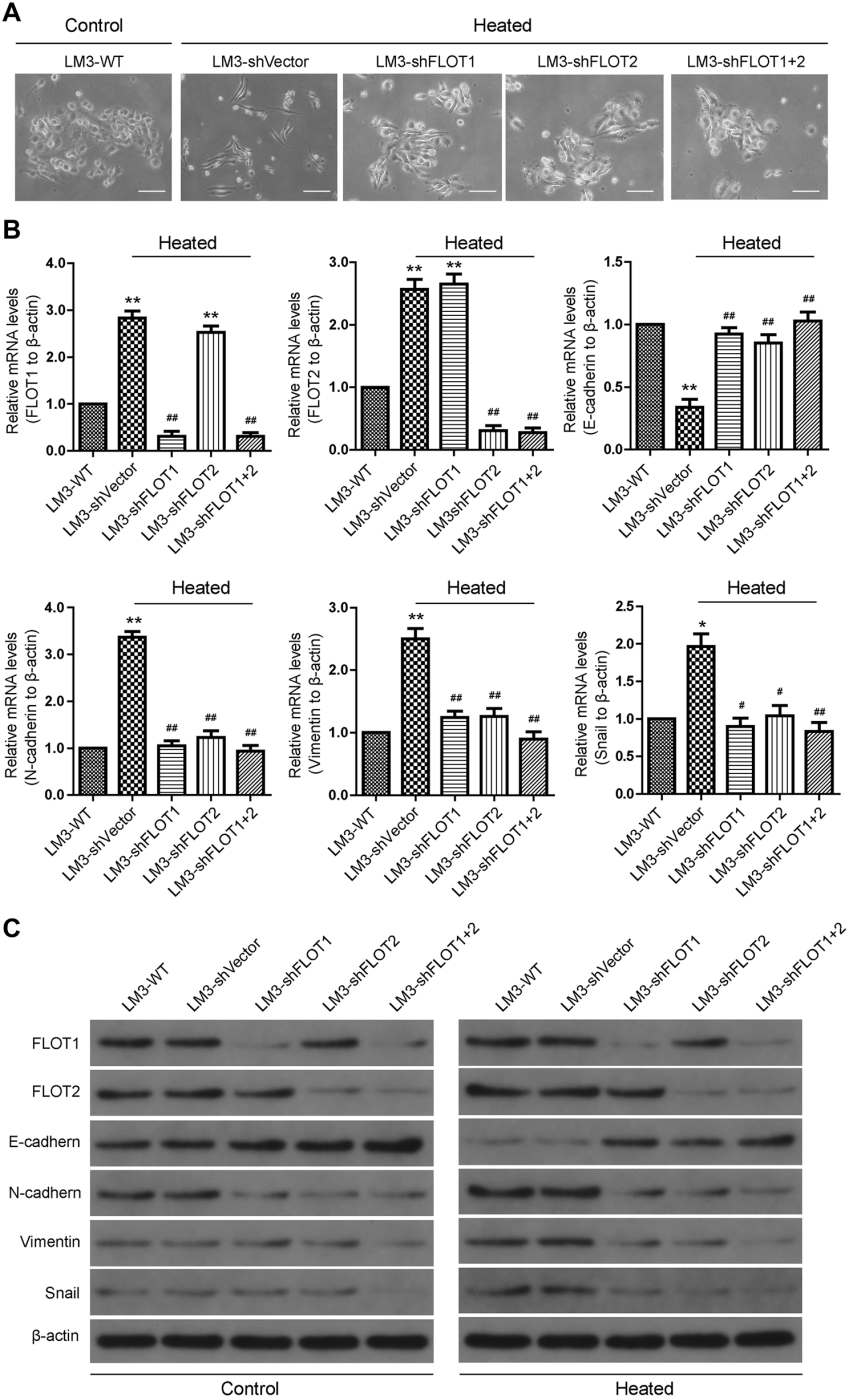
Silencing FLOT1 and FLOT2 attenuated EMT-like changes in heat-treated HCCLM3 cells in vitro. **a** Morphologic changes observed in untreated and heat-treated HCCLM3 cells that were transfected with shVector, shFLOT1, shFLOT2, or shFLOT1 + 2. **b, c** RT-qPCR and western blot analyses of FLOT1, FLOT2, E-cadherin, *N*-cadherin, Vimentin, and Snail expression in control and heat-treated HCCLM3 cells that were transfected with shVector, shFLOT1, shFLOT2, or shFLOT1 + 2; scale bar = 50 µm. Data are presented as mean ± SD. Experiments were independently conducted three times; *P < 0.05, **P < 0.01 vs. the HCCLM3-WT group; ^#^*P* < 0.05, ^##^*P* < 0.01 vs. the heat-treated HCCLM3-shVector group

We further elucidate whether the upregulation of FLOT1 and FLOT2 was functionally associated with the EMT-like changes in heat-treated HCCLM3 cells. At 48 h after 45 °C heat treatment, HCCLM3 cells showed an irregular fibroblast-like shape instead of their typical epithelial/cobblestone appearance (Fig. 4a). Meanwhile, RT-qPCR (Fig. 4b) and western blot (Fig. 4c) results demonstrated that E-cadherin expression (epithelial marker) was reduced in heat-treated cells compared with controls, while *N*-cadherin, Vimentin, and Snail were all increased. Silencing FLOT1 and FLOT2 influenced cell morphology and the expression of EMT markers. RT-PCR and western blot data showed that *N*-cadherin, Vimentin, and Snail were decreased in heat-treated HCCLM3-shFLOT1-, HCCLM3-shFLOT2- and HCCLM3-shFLOT1 + 2-expressing cells compared with heat-treated HCCLM3-shVector cells (Fig. 4a–c). Furthermore, the silencing FLOT1 or FLOT2 or both also reduced the expression of *N*-cadherin, vimentin and Snail in HCCLM3 cultured in 37 °C (Supplemental Fig. 3).

### The Akt/Wnt/β-catenin pathway plays a vital role in the change of metastatic potential of heat-treated HCCLM3 cells in vitro

**Fig. 5:**
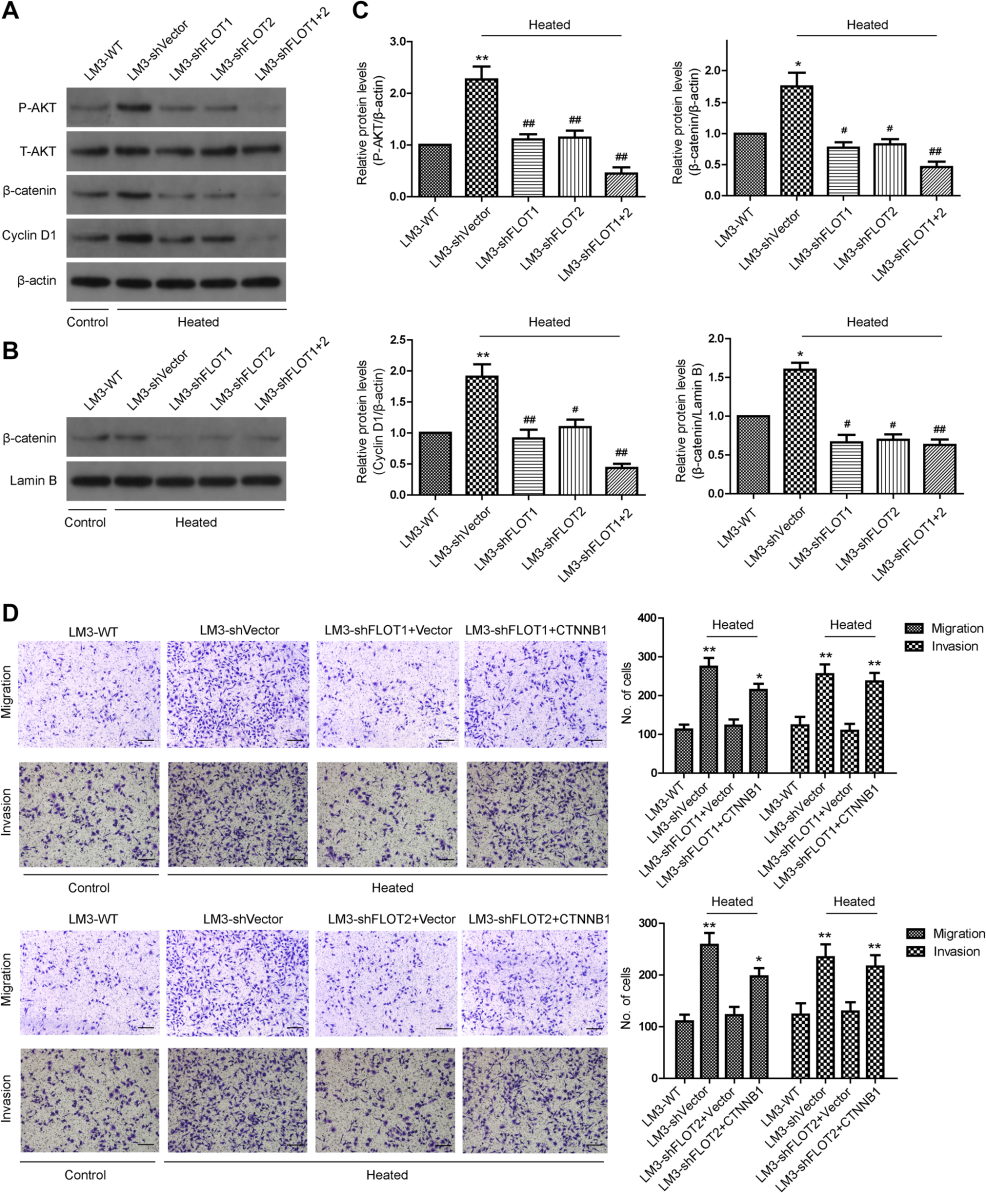
The Akt/Wnt/β-catenin pathway plays a vital role in the increased metastatic potential of residual HCCLM3 cells after insufficient RFA. **a** Western blot analysis of phosphorylated (p)Akt, and total β-catenin and Cyclin-D1 in HCCLM3 cells that were transfected with shVector, shFLOT1, shFLOT2, or shFLOT1 + 2. **b** Nuclear distribution of β-catenin in HCCLM3 cells was detected by western blot. **c** Densitometry showing relative changes in FLOT1 and FLOT2 expression. **d** Representative images of migration and invasion assays of HCCLM3 cells transfected with shVector, shFLOT1, and shFLOT2 ± CTNNB1 were analyzed using transwell assays; scale bar = 100 µm. Data are presented as mean ± SD. Experiments were independently conducted three times; *P < 0.05, **P < 0.01 vs. heat-untreated HCCLM3-WT group; ^#^*P* < 0.05, ^##^*P* < 0.01 vs. heat-treated HCCLM3-shVector group

We previously found that insufficient RFA enhanced the invasive and metastatic potential of residual HCCLM3 cells by activating β-catenin signaling (Zhang et al. 2014a). Therefore, in this study, we investigated whether β-catenin signaling is required for the FLOT1- and FLOT2-mediated increase in HCCLM3 aggressiveness. As shown in Fig. 5a, c, levels of phosphorylated (p)Akt, β-catenin and Cyclin-D1 were increased in heat-treated HCCLM3-shVector cells. Additionally, a marked translocation of intracellular translocation β-catenin in heat-treated HCCLM3-shVector cells was verified by western blot (Fig. 5b, c). In contrast, the phosphorylation level of AKT and the expression of β-catenin and Cyclin-D1 were decreased in the HCCLM3-shFLOT1, HCCLM3-shFLOT2 as well as HCCLM3-shFLOT1 + 2 cells (Fig. 5a–c). Similarly, the silencing of FLOT1 or FLOT2 or both reduced the expression of phosphorylation level of AKT, cytoplasmic and nuclear β-catenin in HCCLM3 cultured in 37 °C (Supplemental Fig. 4). Moreover, overexpressing β-catenin restored the increased migration and invasion following heat treatment of HCCLM3-shFLOT1 and HCCLM3-shFLOT2 cells (Fig. 5d). Taken together, these results demonstrate that AKT/β-catenin/Cyclin D1 signaling is essential for FLOT1 and FLOT2-induced migration and invasion in heat-treated HCCLM3 cells.

### Silencing FLOT1 and FLOT2 inhibited tumor growth and EMT of HCCLM3 cells in vivo

**Fig. 6:**
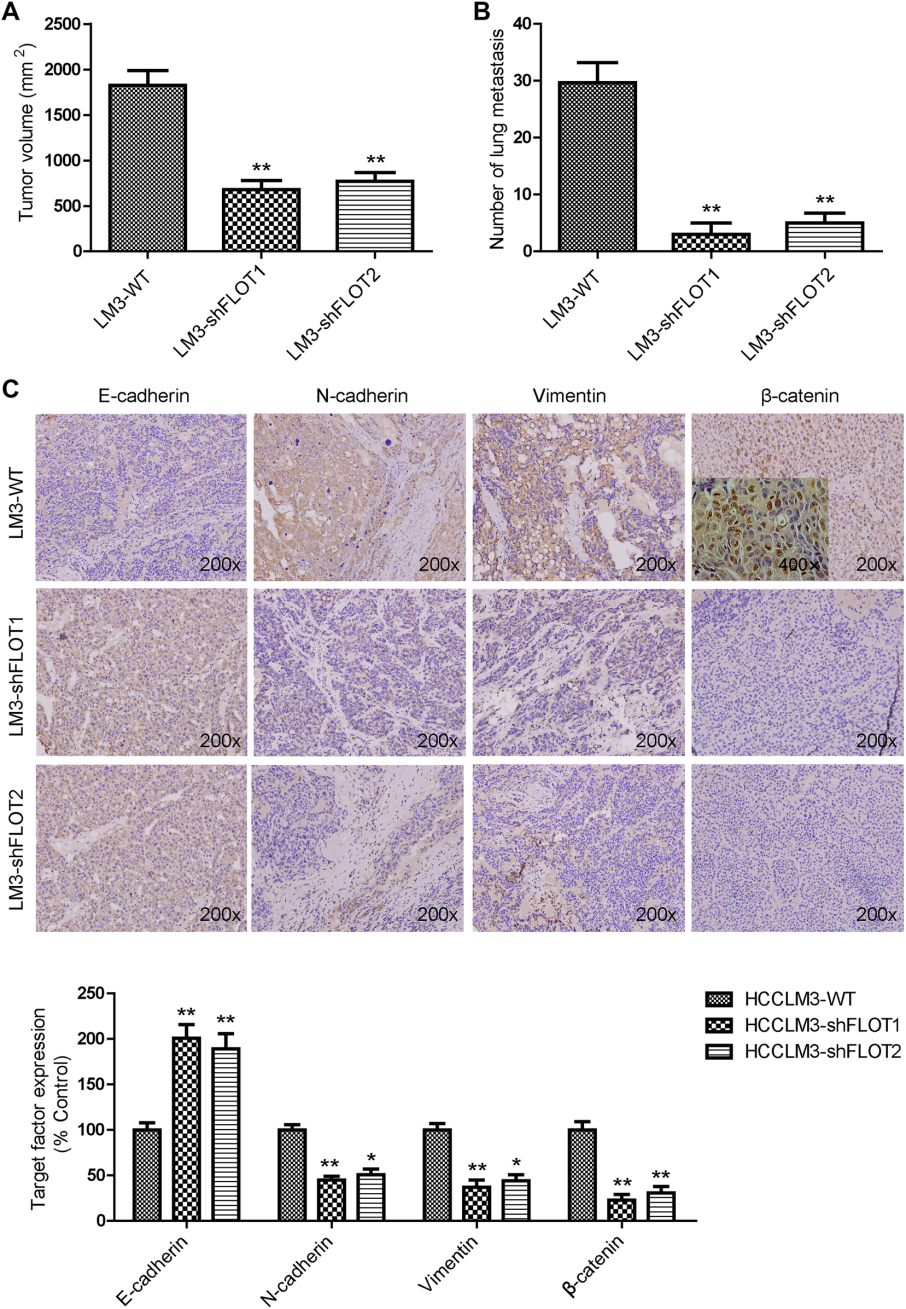
Silencing FLOT1 and FLOT2 inhibited growth and EMT of HCCLM3 cells in vivo. **a** Tumor sizes in the HCCLM3-LV-shFLOT1 and HCCLM3-shFLO2 groups were smaller than in the HCCLM3-WT group. **b** Silencing FLOT1 and FLOT2 inhibit the lung metastasis of HCCLM3 residual cancer after insufficient RFA. **c** Immunohistochemistry revealed that E-cadherin expression was decreased, while *N*-cadherin, Vimentin and β-catenin expression were increased in HCCLM3-shFLOT1 and HCCLM3-shFLOT2 tumors compared with HCCLM3-controls. Representative images are shown at 200× magnification. Data are presented as mean ± SD. Experiments were independently conducted three times; *P < 0.05, **P < 0.01 vs. the HCCLM3-WT group; ^#^*P* < 0.05, ^##^*P* < 0.01 vs. heat-treated HCCLM3-shVector group

Finally, we determined whether FLOT1 and FLOT2 influenced the behavior of residual tumor cells after insufficient RFA intervention in vivo. In insufficient RFA group, the tumor volume of HCCLM3-shVector was significantly larger than those in the control group (1476.7 ± 95.1 vs. 1827.2 ± 161.7 mm^3^, *P* < 0.05; Fig. 6a). Either HCCLM3-shFLOT1 or HCCLM3-shFLOT2 tumors were smaller than HCCLM3-shVector tumors in the control mice (575.7 ± 90.3 vs. 1476.7 ± 95.1 mm^3^, *P* < 0.01; 650.1 ± 91.9 vs. 1476.7 ± 95.1 mm^3^, *P* < 0.01) or insufficient RFA-treated mice (680.7 ± 97.8 vs. 1827.2 ± 161.7 mm^3^, *P* < 0.01; 769.3 ± 98.49 vs. 1827.2 ± 161.7 mm^3^, *P* < 0.01; Fig. 6a). To further evaluate the metastatic potential of residual cancer of HCCLM3 after insufficient RFA, serial lung paraffin sections were used. The pulmonary metastases in insufficient RFA group were significantly increased, compared with the control group; while FLOT1 or FLOT2 knockout can inhibit the pulmonary metastases in the control or insufficient RFA-treated mice (Fig. 6b). We further examined E-cadherin, *N*-cadherin, Vimentin, and β-catenin expression in orthotopic tumor samples after insufficient RFA. Immunohistochemistry revealed typical membranous E-cadherin expression at cell–cell contacts in the HCCLM3-shFLOT1 and HCCLM3-shFLOT2 groups, and low *N*-cadherin, Vimentin and β-catenin expression. In contrast, HCCLM3-shVector tumors showed significantly lower E-cadherin expression, and higher *N*-cadherin, Vimentin, and β-catenin expression (Fig. 6c). These results demonstrated that FLOT1 and FLOT2 promoted residual HCC tumor growth and metastasis after insufficient RFA in vivo.

## Discussion

In this study, we first found that FLOT1 and FLOT2 were overexpressed in HCC tissues after insufficient RFA and in HCC cell lines after heat treatment. Next, we explored the role of FLOT1 and FLOT2 in HCC growth and metastasis in vivo and in vitro. In accordance with studies in other cancers (Berger et al. 2013; Song et al. 2012; Wang et al. 2013), knocking down FLOT1 and/or FLOT2 inhibited the invasion, migration, and anchorage-independent growth of HCCLM3 cells in vitro and decreased their tumor growth and metastasis in vivo.

Flotillins encode caveolae-associated integral membrane proteins that belong to the lipid raft family, and are involved in vesicular trafficking and signal transduction(Bickel et al. 1997). The roles of flotillins in cancer progression have been studied in various cancers (Gao et al. 2015; Li et al. 2014; Zhang et al. 2014b). Flotillin overexpression promotes the proliferation, invasion, migration, and metastasis of cancer cells. Our previous studies had demonstrated that thermal intervention can enhance the metastasis of residual cancer by activating the FAK/PI3k signaling pathway (Zhang et al. 2017). A recent research pointed out that overexpression of flotillin can interact with α-actin and affect the activity of FAK, thereby enhancing the migration and metastasis of cancer(Banning et al. 2018). Whether thermal interference can affect the expression of flotillin is worth exploring. The present study confirmed for the first time that the expression of flotillin was up-regulated in residual cancer tissues after thermal interference.

In this study, we found that insufficient RFA increased both FLOT1 and FLOT2 in HCCLM3 cells but not in HepG2 cells, indicating these human HCC cell lines have different biological characteristics. Inversely, knocking down either FLOT1 or FLOT2 almost completely blocked the enhanced aggressiveness of heat-treated HCCLM3 cells in vitro and of residual cancer after insufficient RFA in vivo. These findings indicated a prominent role for flotillins in insufficient RFA-induced metastasis.

EMT is a biological process through which polarized epithelial cells undergo phenotypic changes into mesenchymal cells with enhanced invasiveness, survival, and extracellular matrix production(Kalluri and Weinberg 2009). Furthermore, EMT contributes to metastasis in a variety of human cancers (Hugo et al. 2007; Yeung and Yang 2017). Several studies have demonstrated that suboptimal RFA accelerated HCC growth and spread by transiently inducing an EMT-like and more aggressive cellular phenotype (Iwahashi et al. 2016; Yoshida et al. 2013). Meanwhile, the EMT-related transcript factors snail and twist were significantly higher in RFA group than those in the non-RFA group in clinical HCC cases (Iwahashi et al. 2016; Zeng et al. 2018). Herein, we showed that silencing FLOT1 and FLOT2 increased E-cadherin expression and decreased *N*-cadherin, Vimentin and Snail in heat-treated HCCLM3 cells. Finally, these changes were also found in xenografts in nude mice after insufficient RFA. Thus, this study provides new insights into the mechanisms through which EMT is regulated in residual HCC after insufficient RFA and suggests FLOT1 and FLOT2 as potential targets for anti-metastatic therapy.

Multiple signaling pathways regulate EMT, but considering the central role of Wnt/β-catenin in HCC biology and the well-characterized association between Wnt/β-catenin signaling and EMT, we focused on the Wnt/β-catenin pathway (Dahmani et al. 2011; Monga 2015; Waisberg and Saba 2015). In this study, the effects of flotillins on β-catenin expression were examined, and elevated total and intra-nuclear β-catenin levels were verified in HCCLM3 cells in vitro. The key downstream gene of Wnt/β-catenin signaling, Cyclin-D1, was also upregulated. Moreover, we also detected pAKT levels because AKT phosphorylation can enhance β-catenin nuclear accumulation (Fang et al. 2007). Moreover, AKT signaling pathway is closely related to EMT in HCC, colorectal cancer, gastric cancer and breast cancer (Duan et al. 2018; Huang et al. 2018; Jiang et al. 2019; Zhang et al. 2018). Li et al. found that up- and downregulation of FLOT1 remarkably affected cervical cancer cell motility and invasion, respectively, through the EMT (via Wnt/β-catenin) and NF-κB pathways (Li et al. 2016). Liu et al. showed that FLOT2 promoted metastasis in nasopharyngeal carcinoma by activating the NF-κB and PI3K/Akt3 pathways (Liu et al. 2015). In bladder cancer, up-regulation of FLOT1 could also reverse the suppressed cell proliferation caused by miR-608 via activating AKT signaling (Liang et al. 2017). Additionally, as a direct target of miR-133, FLOT2 was regulated via Akt signaling and played pro-metastatic role in lung adenocarcinoma cell (Wei et al. 2018). Our results showed that pAKT was increased by heat treatment and decreased by silencing FLOT1 and/or FLOT2. Conversely, overexpressing β-catenin rescued the increase in motility and invasion of HCCLM3 cells following heat treatment that was lost when FLOT1 or FLOT2 were knocked out. Our findings demonstrate that insufficient RFA enhances the invasion and metastasis of residual cancer cells via Akt/Wnt/β-catenin-regulated EMT.

In summary, this study demonstrated that insufficient RFA enhanced the metastatic ability of HCC cells in vivo and in vitro, which was mediated by Akt/Wnt/β-catenin signaling. Furthermore, insufficient RFA increased FLOT1 and FLOT2 expression, suggesting these lipid raft protein promote these signaling pathways. Therefore, future studies will attempt to decrease the invasion and metastasis of residual cancer cells to improve the curative effects of RFA in HCC patients.

## Electronic supplementary material

Below is the link to the electronic supplementary material.

Supplementary material 1 (TIF 7917 KB)

Supplementary material 2 (TIF 7414 KB)

Supplementary material 3 (TIF 1746 KB)

Supplementary material 4 (TIF 1429 KB)
