# Supplementary figures and images for: Insufficient radiofrequency ablation promotes the metastasis of residual hepatocellular carcinoma cells via upregulating flotillin proteins
Source: J Cancer Res Clin Oncol. 2019 Feb 28;145(4):895–907. doi: 10.1007/s00432-019-02852-z (PMC6435628; doi:10.1007/s00432-019-02852-z)

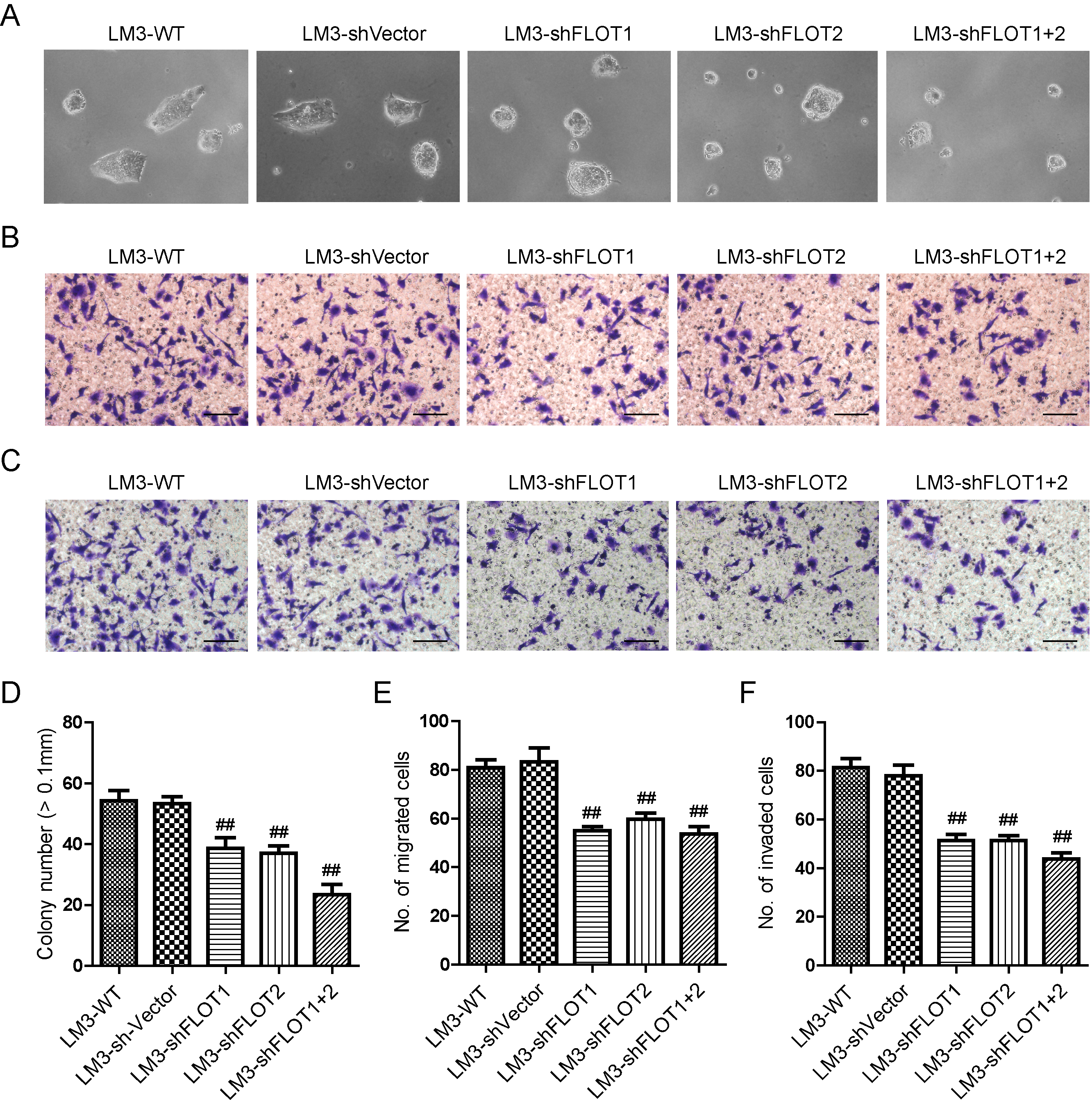

Supplement: Supplementary file 1 — Supplementary material 1 (TIF 7917 KB) [file 432_2019_2852_MOESM1_ESM.tif]

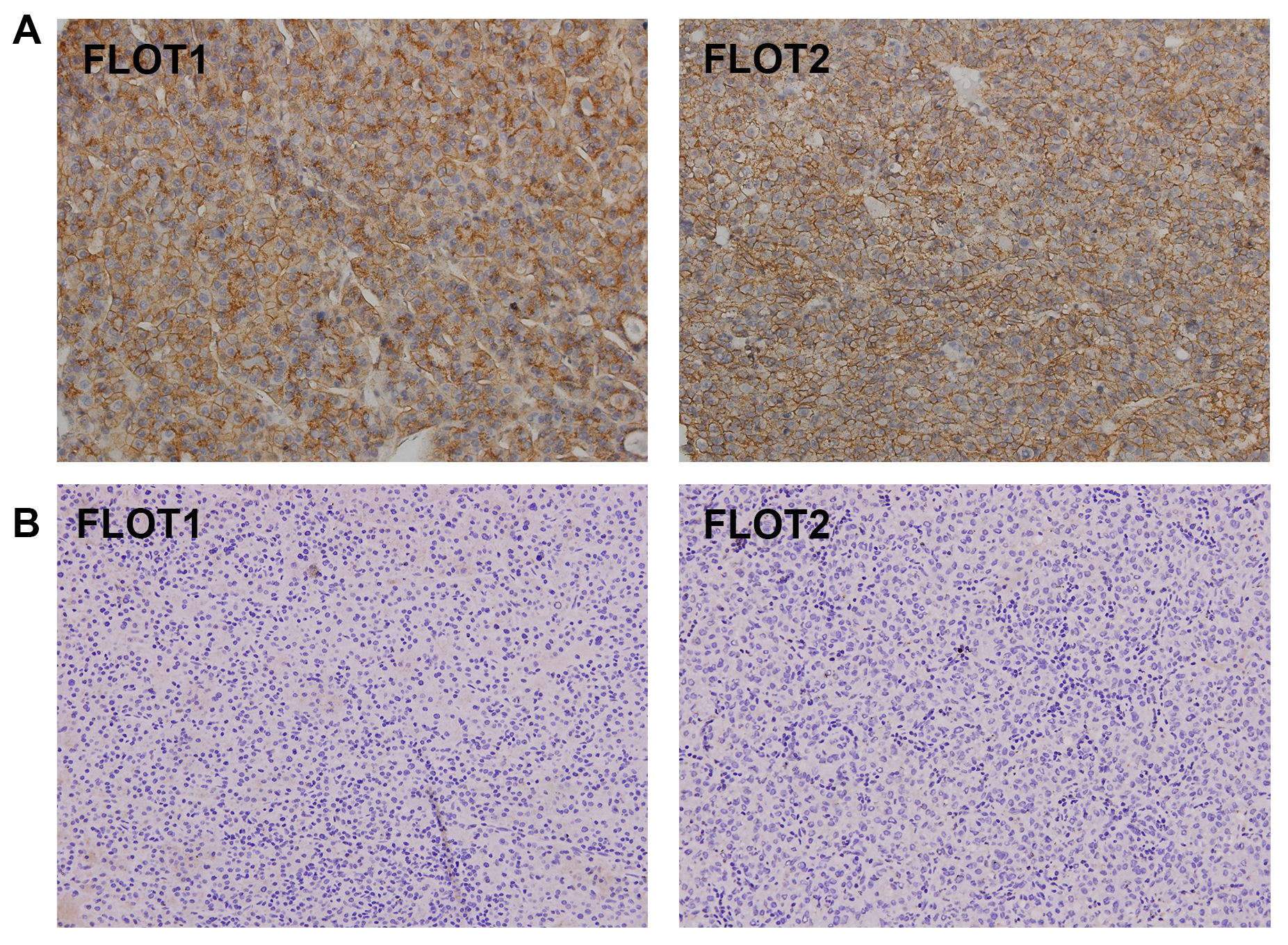

Supplement: Supplementary file 2 — Supplementary material 2 (TIF 7414 KB) [file 432_2019_2852_MOESM2_ESM.tif]

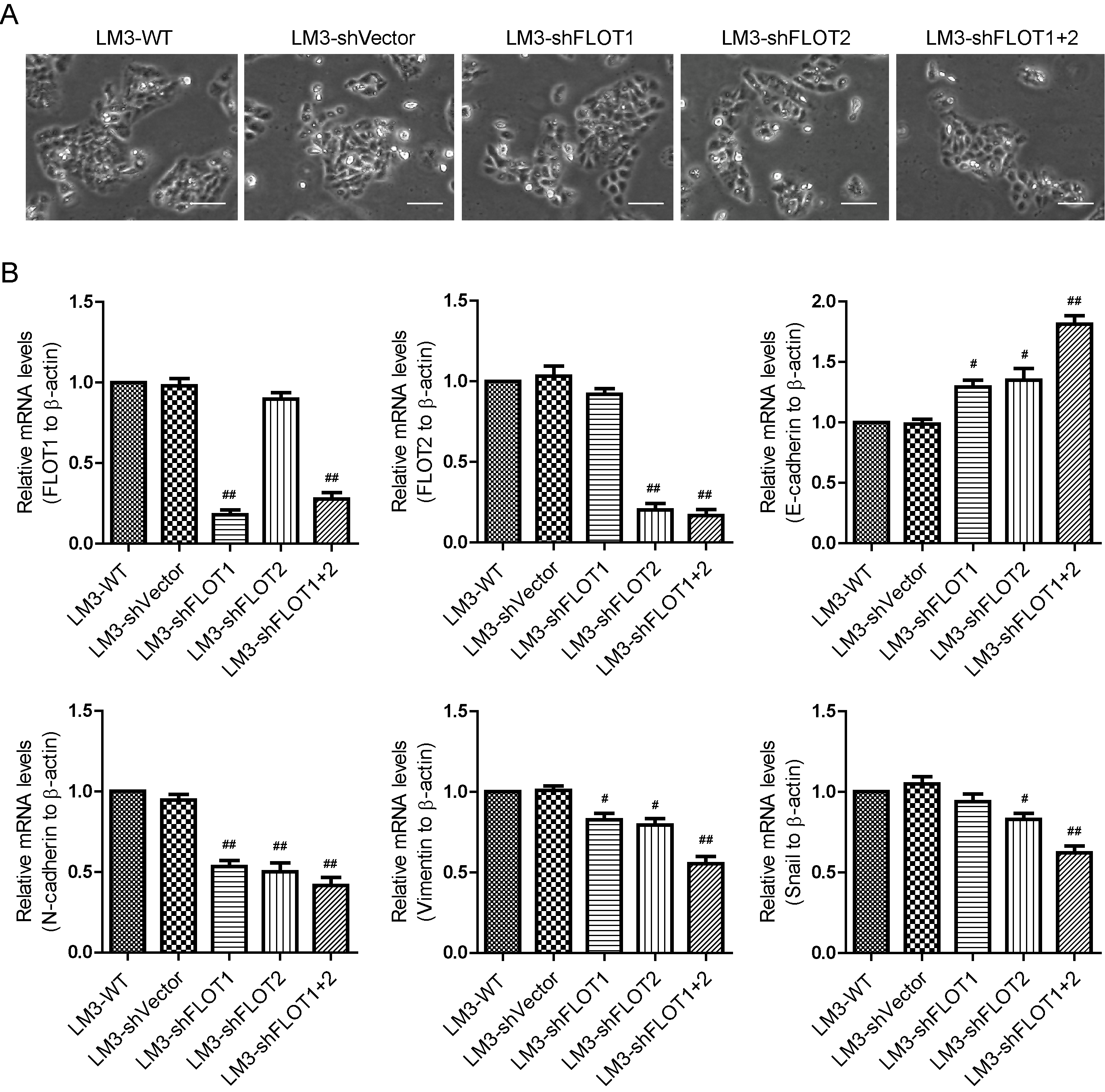

Supplement: Supplementary file 3 — Supplementary material 3 (TIF 1746 KB) [file 432_2019_2852_MOESM3_ESM.tif]

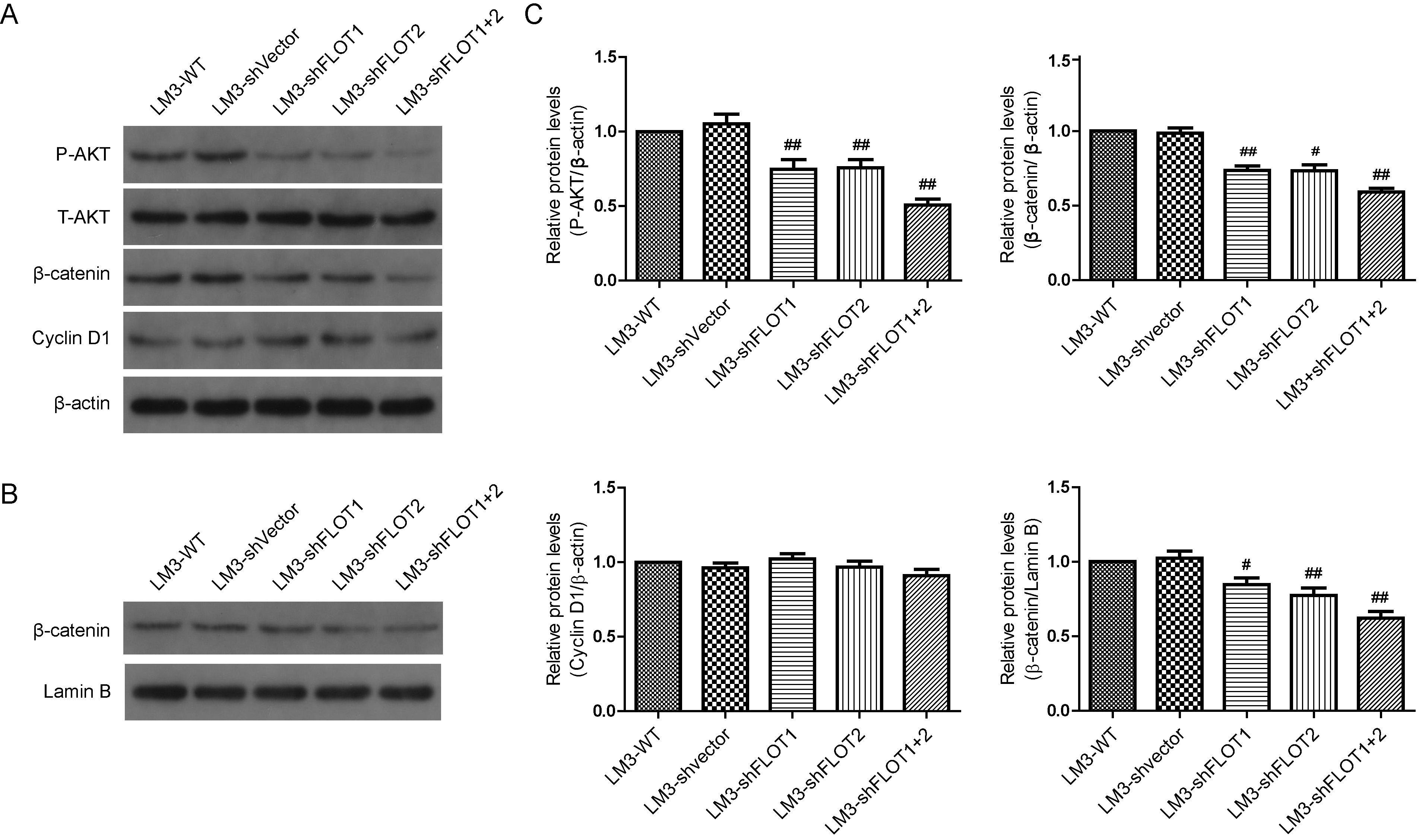

Supplement: Supplementary file 4 — Supplementary material 4 (TIF 1429 KB) [file 432_2019_2852_MOESM4_ESM.tif]
